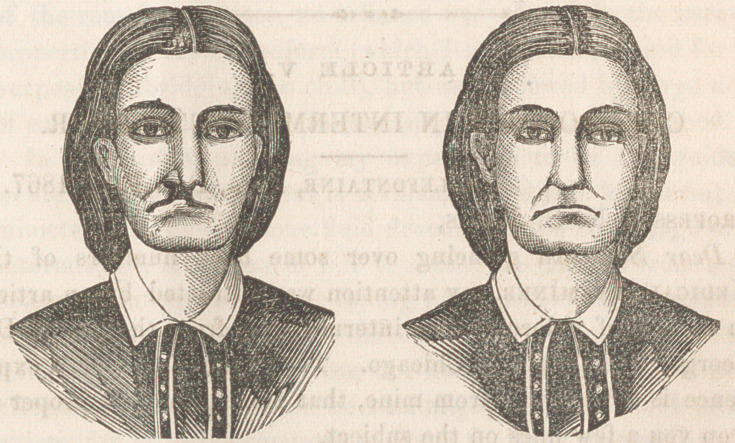# Result of Operation for Harelip

**Published:** 1868-02

**Authors:** S. D. Mercer

**Affiliations:** Omaha, Nebraska


THE
CHICAGO MEDICAL EXAMINER.

N. S. DAVIS, M.D., EDITOR.

VOL. IX. FEBRUARY, 1868. NO. 2.

### Original Contributions.

ARTICLE IV.

### RESULT OF OPERATION FOR HARELIP.

BY S. D. MERCER, M.D, Omaha, Nebraska.

Miss Harriet L., *æt.* 30, of Omaha, consulted me last March, concerning an operation which she said had been frequently declined, owing to the condition of her general health and the extent of the cleft. For several years she had been troubled with dyspepsia, depending upon inefficient mastication, and from the resulting debility, the lungs had become so irritable that the exhibition of an anæsthetic was very difficult.

The above cut represents a deep cleft, of the most unsightly character, rendering the patient an object of disgust to herself and all around her. The chasm extended through the superior maxillary its whole length, the palate bones, and the soft palate, forming a perfect communication between the mouth and left nares. The left side of the nose was very much flattened by contraction of the tissue to the left of the chasm to which the left ala was attached.

By the assistance of Drs. Babcock and Canfield, the patient was partially anæsthetized, and, with the bone forceps, the side of both superior maxillaries bordering on the cleft was removed, the edges of the soft parts pared, an incision made from the septum nari to the base of the right ala, and thence about one-half inch toward the external canthus of the right eye, and another from the base of the left ala toward the external canthus of the left eye. Both sides were then freely dissected up, and the margins united with three pins. The wound united, partly by first intention, but some traumatic erysipelas occurred, followed by very slight suppuration, but the union was subsequently completed by second intention.

The food was subsequently masticated better, and, consequently, the dyspeptic symptoms are gradually disappearing. The patient was referred to the dentist for further improvement.

## Figures and Tables

**Figure f1:**